# Citrus Tree Segmentation from UAV Images Based on Monocular Machine Vision in a Natural Orchard Environment

**DOI:** 10.3390/s19245558

**Published:** 2019-12-16

**Authors:** Yayong Chen, Chaojun Hou, Yu Tang, Jiajun Zhuang, Jintian Lin, Yong He, Qiwei Guo, Zhenyu Zhong, Huan Lei, Shaoming Luo

**Affiliations:** 1 Academy of Contemporary Agriculture Engineering Innovations, Zhongkai University of Agriculture and Engineering, Guangzhou 510225, China; 441200923@163.com (Y.C.); houchaojun@zhku.edu.cn (C.H.); zhuangjiajun@zhku.edu.cn (J.Z.); linjtian@163.com (J.L.); smluo@gdut.edu.cn (S.L.); 2 College of Biosystems Engineering and Food Science, Zhejiang University, Hangzhou 310058, China; yhe@zju.edu.cn; 3 Guangdong Key Laboratory of Modern Control Technology, Guangdong Institute of Intelligent Manufacturing, Guangzhou 510070, China; zy.zhong@giim.ac.cn (Z.Z.); huan.l@giim.ac.cn (H.L.)

**Keywords:** agricultural unmanned aerial vehicles, monocular computer vision, tree crown segmentation, circumstance brightness, weed environment orchard

## Abstract

The segmentation of citrus trees in a natural orchard environment is a key technology for achieving the fully autonomous operation of agricultural unmanned aerial vehicles (UAVs). Therefore, a tree segmentation method based on monocular machine vision technology and a support vector machine (SVM) algorithm are proposed in this paper to segment citrus trees precisely under different brightness and weed coverage conditions. To reduce the sensitivity to environmental brightness, a selective illumination histogram equalization method was developed to compensate for the illumination, thereby improving the brightness contrast for the foreground without changing its hue and saturation. To accurately differentiate fruit trees from different weed coverage backgrounds, a chromatic aberration segmentation algorithm and the Otsu threshold method were combined to extract potential fruit tree regions. Then, 14 color features, five statistical texture features, and local binary pattern features of those regions were calculated to establish an SVM segmentation model. The proposed method was verified on a dataset with different brightness and weed coverage conditions, and the results show that the citrus tree segmentation accuracy reached 85.27% ± 9.43%; thus, the proposed method achieved better performance than two similar methods.

## 1. Introduction

In recent years, fully autonomous agricultural unmanned aerial vehicles (UAVs)were widely applied in orchards, and target detection (e.g., of fruit trees) constitutes one of the key technologies for autonomous operation [1,2,3]. However, unstructured factors (e.g., brightness condition (BC) and weed coverage condition (WCC)) in a complex orchard environment affect the fruit tree detection accuracy [4,5,6,7,8]. Currently, methods employed for the detection of fruit trees with agricultural UAVs consist mainly of spectral imaging and machine vision technologies; as such, the accuracy of these fruit tree detection methods depends greatly on the tree segmentation accuracy [9,10,11].

Many detailed research studies were performed to improve tree segmentation accuracy. Csillik et al. [12] segmented fruit tree crowns with a convolutional neural network (CNN) and a simple linear iteration classification algorithm and detected citrus trees in complex agricultural environments within spectral citrus image datasets obtained with a multi-spectral camera onboard a UAV. Johansen et al. [13] segmented canopies of trees based on a three-dimensional (3D) model containing the height, geometry, and size of the trees and evaluated the pruning effect of crops in multi-spectral images collected by a multi-spectral UAV system on ornamental plants at multiple altitudes. Srestasathiern et al. [14] segmented oil palm trees using a vegetation index through a non-maximum suppression algorithm and monitored the number of trees in high-resolution spectral images. Roope et al. [11] segmented separate spruce trees by establishing a 3D hyper-spectral tree model with hyper-spectral UAV images and a watershed algorithm and estimated the degree of damage wrought by longhorn beetles at the single-tree level with an estimation spectral curve. Malambo et al. [15] segmented crop canopies by establishing a 3D structure from motion (SfM) model with high-resolution UAV images and evaluated corn height parameters in the field. Torres-Sanchez et al. [16] segmented a planted forest by generating a 3D ground model and automatically monitored the forest by extracting geometric parameters through an object-based extraction method in a high-resolution UAV imaging system. Juan et al. [17] segmented individual tree canopies in a digital surface model reconstructed by SfM technology and estimated the canopy position, height, and diameter. Guo et al. [18] segmented a forest canopy in a geometric point cloud model established by a UAV–light detection and ranging (LiDar) system and captured the forest canopy height, canopy coverage and terrestrial biomass of three ecosystems, namely, coniferous broad-leaved mixed, evergreen broad-leaf, and mangrove forests. Pedro et al. [19] segmented chestnut trees in image data from a red/green/blue (RGB) + infrared camera UAV system through an object clustering extraction method and automatically monitored the trees according to their geometric features and the canopy coverage rate of the resulting area. Wu et al. [20] segmented the area of trees with a watershed method and a polynomial fitting method in a 3D forest model established by a UAV–LiDar system and calculated the canopy coverage of a planted forest with a canopy height model and multiple linear regression model. Li et al. [21] segmented the crowns of separate trees in a geometric point cloud image with a 3D forest model established by an airborne laser scanner and achieved the maintenance and management of forest ecology with a global navigation satellite system and an inertial measurement unit auxiliary structure with SfM technology. Omair et al. [22] segmented trees on urban grass with color parameters, a gray-level co-occurrence matrix (GLCM) parameter and a clustering algorithm after eliminating lens distortion and counted the trees automatically with an RGBUAV camera; the segmentation technique achieved an accuracy of 70% and demonstrated good applicability for estimating forest degradation. Maciel et al. [23] detected the center line of citrus trees in a high-density orchard with a CNN algorithm in a sliding window and segmented single trees with a CNN; the results showed an overall accuracy of 94% in seven different test orchards. Ramesh et al. [24] segmented single fruit trees with an extreme learning machine, a geometry filtering threshold, and a watershed separation algorithm and detected the numbers of banana, mango, and coconut trees in different orchards with high-resolution RGB cameras onboard a fixed-wing UAV and a multi-rotor UAV with an accuracy of 85%. Lin et al. [25] segmented the areas of single-tree canopies in oblique UAV images with k-means clustering in the La*b* color space (defined by the International Commission on Illumination (CIE) in 1976) and a threshold method with pseudo-NDVI color mapping and texture mapping technology. Carlos et al. [26] applied four segmentation methods (k-means, artificial neural network (ANN), random forest (RForest), and spectral indices (SI)) to grape tree canopies and found that the SI+ANN and RForest methods were superior, with an accuracy of approximately 0.98 in high-resolution UAV images of trees under different shade and soil conditions, useful for the exquisite management of commercial vineyards. Zhao et al. [27] segmented regions of pomegranate trees using the U-net and a region-CNN with a high-resolution visible imaging and multi-spectral imaging UAV system and calculated the water stress parameter and nutritional status of the trees in multi-spectral data. Ultimately, they found that the region-CNN provided better segmentation results. Corey et al. [28] segmented seedling trees in cut-down forest images acquired by an automatically controlled UAV with a chromatic aberration segmentation algorithm in combination with a vertical take-up and take-off algorithm and satellite positioning technology. In addition, they counted the number of seedling trees using a classification and regression tree machine learning model to estimate the forest regeneration rate. Sabzi et al. [29] developed a computer vision method to detect apples in trees and estimated their ripeness based on the most effective color features and ANN classifier; the proposed method achieved an accuracy of 97.88%. Li et al. [30] presented a corn classification method based on computer vision and the maximum-likelihood estimator for classifying normal and damaged corn, and the results showed an overall accuracy of 96.67%.

Although many tree segmentation methods were studied for UAV images in recent years, those methods were mainly applied in structured environments. In contrast, the use of segmentation methods in unstructured environments, especially in citrus orchards under different brightness and weed coverage conditions (e.g., some weeds in a citrus orchard can improve the physical and chemical properties of the soil and attract beneficial insects [31]), is yet to be reported. Considering the cost of equipment, the coverage of the algorithm, the detection accuracy, and the portability of the system, this paper proposes a citrus tree segmentation method based on monocular machine vision in a natural orchard environment. Therefore, the main processes used to segment citrus trees in unstructured environments were as follows: (1) development of an illumination compensation algorithm to pre-process the under-lit foreground in citrus orchard images, thereby reducing the sensitivity of the algorithm to the environmental brightness, (2) extraction of the potential regions of interest (RoIs, i.e., the citrus trees) from weed elements as accurately as possible by combining chromatic aberration technology and the Otsu threshold method, and (3) accurate segmentation of trees from orchard images with a binary detection support vector machine (SVM) established based on the calculated values of 14 color features, five texture features, and local binary pattern (LBP) features.

## 2. Materials

To debug and test the proposed method, a monocular RGB camera was outfitted on an agricultural UAV and used to acquire image datasets with in the height range of the agricultural UAV during operation. The equipment and the data are briefly introduced in this section.

### 2.1. The Study Area and the UAV Equipment

**Figure 1:**
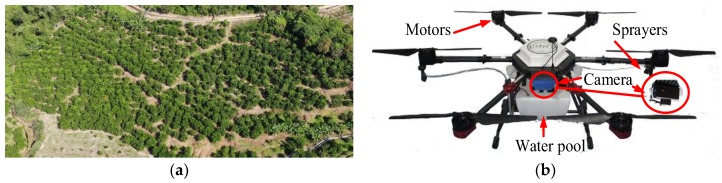
The general view of the orchard and the physical photo of the unmanned aerial vehicle (UAV). (**a**) General view of the citrus orchard captured by a UAV with an operation height of 100 m. (**b**) The physical photo of the agricultural UAV.

The study area is a commercial orange orchard of Conghua Hualong Fruit and Vegetable Preservation Co., LTD, located in Conghua District in Guangzhou, China (113°31′15.5″ east (E), 23°38′42.4″ north (N)). The orchard covers an area of 0.6 km^2^, and the center of its terrain is 8.9 m higher than its edge. There are 453 “Shatangju citrus” (*Citrus reticulata* Blanco) trees with tree ages of 4.5 years in the orchard. Furthermore, the tree height is 2.7 ± 0.9 m, and the crown diameter is 2.8 ± 0.8 m (minimum diameter of 0.9 m and maximum diameter of 3.4 m). An overall image of the orchard is shown in Figure 1a.

An agricultural UAV developed in the laboratory of Zhongkai University of Agriculture and Engineering (ZHKU0606-1510-CS) was used to collect the dataset of orchard images of the study area. As shown in Figure 1b, the UAV structure includes a kinetic section, an image collection section, and a spraying section. In particular, the kinetic section provides the impetus for flight movement and carrying equipment and consists of six high-speed motors, a control circuit, and a control module. The kinetic section provides a horizontal positioning accuracy of 0.5 m and a vertical positioning accuracy of 0.1 m when controlled by a global positioning system (GPS) unit and remote controller. The onboard image collection section consists of a high-resolution RGB camera (Hawkeye Firefly 8SE Action), which saves images on a secure digital memory card and is fixed by an angle fixer that reduces the high-frequency noise generated by the six high-speed motors. The spraying section, which will be used for future spraying tests, includes nozzles, a water pool, a pump, and pipes.

Considering the spraying height [32], the topographic relief of the orchard, and the height of the fruit trees, the camera distance for the UAV was set to 10–15 m. During each image acquisition process, the image collection section was set vertically downward, and the UAV was suspended steadily in the air until each image was fully collected. The data were stored as joint photographic expert group (JPEG) format to implement a debugging algorithm on a personal computer (PC). The parameters of the UAV and the onboard camera are shown in Table 1.

### 2.2. The Dataset

The dataset comprised 7148 trees in 334 images (the parent dataset, dataset 0) collected in seven batches from November 2018 to March 2019. The images in the natural orchard environment were classified into six categories, namely, two BC categories, insufficient brightness(IB) and sufficient brightness(SB), and three WCC categories, namely, small weed coverage rate (SWCR), medium weed coverage rate (MWCR), and large weed coverage rate (LWCR).The WCCs were defined as follows: SWCR, *A* < 35%; MWCR, 35% ≤ *A* < 60%; LWCR, *A* ≥ 60%, where *A* is the ratio of the foreground (the area was [π/2, π] in H of the hue/saturation/intensity (HSI) color space) to the image area. Furthermore, IB indicates that *I_A_* < 0.3, whereas SB indicates that *I_A_* ≥ 0.3, where *I_A_* is the average illumination value (I of the HSI color space) of area *A*. Table 2 shows the number of images and the number of fruit trees in each of the six (2 × 3; (IB, SB) × (SWCR, MWCR, LWCR)) categories of the data.

**Figure 2:**
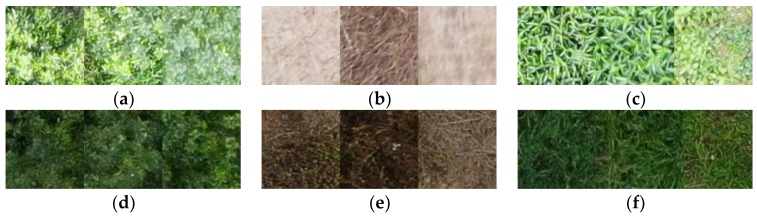
In sufficient brightness (IB) and sufficient brightness (SB) examples of dataset 0. (**a**) Positive area sample of fruit trees under SB conditions. (**b**) Negative area sample of soil or withered grass under SB conditions. (**c**) Negative area sample of weeds under SB conditions. (**d**) Positive area sample of fruit trees under IB conditions. (**e**) Negative area sample of soil or withered grass under IB conditions. (**f**) Negative area sample of weeds under IB conditions.

Half of the data in each category of dataset 0 were randomly selected as the training set, and the remaining images were set aside as the test set. Each training area in the training set was manually marked as rectangles of 200 × 200 pixels, including 1000 positive samples of fruit trees, 200 negative samples of soil or withered grass (in winter or after the application of herbicide), and 800 negative samples of weeds. Figure 2 shows some examples of images within dataset 0 under IB and SB conditions.

The training set and test set described in Section 2.2 were used to train and test the SVM segmentation model. The RoIs of the training set were manually extracted after the illumination compensation; the color and texture features were calculated in the completely marked areas. In addition, the RoIs of the test set were extracted with the method introduced in Section 3.1 and Section 3.2; these RoIs were complete when the color features were calculated, and these RoIs were the maximum inner rectangles when the texture features were calculated.

## 3. The Citrus Tree Segmentation Method

**Figure 3:**
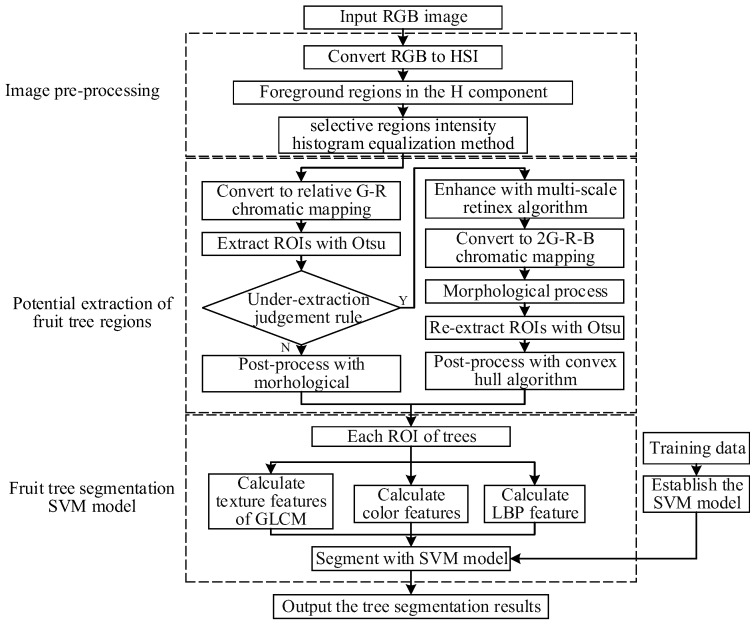
Flow chart of the proposed segmentation method.

Figure 3 shows the three main processes of the method proposed in this paper: image pre-processing, RoI extraction, and fruit tree segmentation. In the image pre-processing stage, the selective region intensity histogram equalization method (SRIHE) was used to reduce the sensitivity of the algorithm to the brightness. Then, to extract the RoIs from the orchard images, chromatic aberration technology was combined with the Otsu method. Finally, the color and texture features of the RoIs were calculated to establish the SVM segmentation model for the citrus trees. The data described in Section 2.2 were used to evaluate the proposed segmentation method. All experiments were performed using MathWorks MATLAB R2018a software on a PC equipped with an Intel(R) Pentium(R) G4600 (3.60 GHz) central processing unit (CPU) and 16 GB of random-access memory (RAM).

### 3.1. Image Pre-Processing

In the orchard images collected by the agricultural UAV, an insufficiently illuminated environment would affect the brightness contrast of fruit trees and reduce the extraction effect of the segmentation method. Therefore, the SRIHE was proposed to pre-process the images, thereby compensating for the brightness contrast of the foreground. The SRIHE calculates the brightness histogram (the I component in the HSI color space) of the image foreground, the areas of which are selected in the H component. The illumination contrast of the foreground is then highlighted and changed to the H and S components of the HSI color space, which is helpful for the subsequent extraction of RoIs and segmentation of fruit trees. Compared to the traditional histogram equalization method in illumination (HE), the SRIHE selects the foreground to compensate for the brightness contrast even when the foreground is in darkness, while the HE takes the global histogram for the adjustment and potentially lacks a successful effect, as it is affected by the background [33], which is consistent with the findings of Tan et al. [34] and Kim et al. [35]

As inputs to the SRIHE, the images were firstly transformed from RGB color space to HSI color space to obtain three independent components: H, S, and I. The range of each pixel in the H component was [0, 2π], reflecting the color of each pixel perceived by the human eye, where the range [π/2, π] denotes the color green. The green area was marked, and the set of pixels in the green range of H was denoted as *S_g_*, which was expressed as $S_{g}=\{\left(s,y\right)|\pi /2\leq H\left(x,y\right)\leq \pi \}$. The histogram of *I_g_* was calculated and equalized to *I_g_’*, where *S_g_* is the I component in HSI color space. The pixels outside of *S_g_* remained and were recombined with *I_g_’* to form a new brightness mapping *I’*. Then, *I’* and the original H and S mapping were compiled to form new HSI’ images, which were then transformed into new RGB images.

### 3.2. Potential Fruit Tree Region Extraction

To extract RoIs from different WCC backgrounds, the RG chromatic extraction method (ERGCM) was proposed to preliminarily extract RoIs from orchard images. Because the ERGCM is strongly sensitive to weeds and under-extracts RoIs from a weedy background, an under-extraction judgement rule (UEJR) was established to judge whether the preliminary result was under-extracted. If so, the under-extracted images would be re-extracted using the chromatic mapping method presented herein, namely, the EMSRCM, which was proposed based on the combination of multi-scale retinex (MSR) and chromatic technology.

#### 3.2.1. RG Chromatic Tree Extraction Method

**Figure 4:**
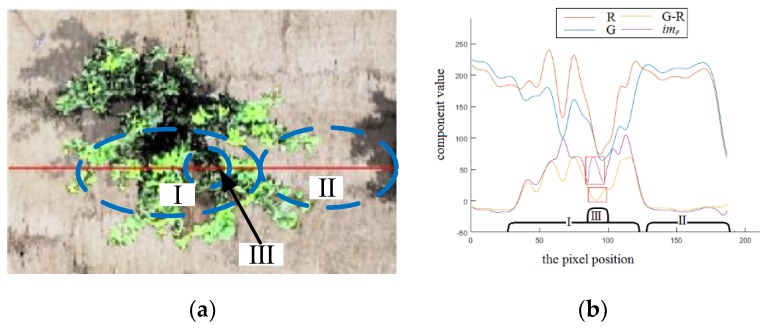
R (the red component in RGB color space), G (the green component), G–R (the traditional G–R chromatic value), and *im_p_* (the relative G–R chromatic value) curves of a tree in a small weed coverage rate (SWCR) image. (**a**) Example of the *I_a_* of a tree in an SWCR image. (**b**) R, G, R–G, and *im_p_* curves of the line in *I_a_*.

Since the trees and background in SWCR images have distinguishable color characteristics, the proposed ERGCM is an appropriate technology for extracting RoIs, as shown in Figure 4. A screenshot of a single tree in an SWCR image is shown in Figure 4a,b, the R curve (the red component in RGB color space), G curve (the green component), G–R curve (the traditional G–R chromatic value), and *im_p_* curve (the relative G–R chromatic value) of each pixel of the marked red line in Figure 4a. Comparing regions **I** and **II** (region **I** is the fruit tree area, and region **II** is the background) of the R and G curves in Figure 4b, the color differences in the R and G values between the trees and background can be quantified, as R is larger than G in **II** and smaller in **I**. Therefore, the G–R value is larger in region **I** than in region **II**, confirming that chromatic mapping is preferable for the extraction of trees in SWCR images. However, in region **III**, the G–R value in region **III** is smaller than that in region **I**; region **III** exhibits a weak local brightness, while region **I** highlights one tree. This might be because the traditional G–R chromatic value is the absolute difference between G and R and is, thus, affected by the local brightness. To reduce the effect of the local brightness, *im_p_*, which is relative to the pixel brightness I (I component in HSI color space), was introduced. The transformation process is expressed by Equation (1).

(1) $$im_{p}=\{\begin{array}{cc} G_{p}-R_{p}/I_{p}, & I_{p}>0\\ 0, & I_{p}=0 \end{array}$$

where *p* is a single pixel in an image, *R_p_* is the R component of pixel *p* after the SRIHE processing step, *G_p_* is the G component of pixel *p*, and *I_p_* is the I component of pixel *p* in HSI color space. Comparing the G–R and *im_p_* curves in region **III** (the curve sections within the two red rectangles in Figure 4b), the G–R value is severely decreased, which might reduce the extraction and segmentation accuracy, but the *im_p_* value is still acceptable. This is because *im_p_* takes the local brightness into account; in addition, the RG chromatic mapping is measured relative to the brightness of the pixels.

In *im_p_* mapping, the illumination of a tree is relatively larger than that of the background, which is obviously different, creating a histogram with a bimodal structure. To minimize the misclassification probability of extracting trees under SWCR conditions, the Otsu method [36] was adopted to calculate the optimal threshold to extract the RoIs (fruit trees) by maximizing the between-cluster variance. Subsequently, to remove areas of small interference outside, inside, and around (at the edges) the RoIs and to improve the RoI extraction accuracy, the morphological method was adopted. Firstly, the small disturbances outside the RoIs might be caused by an impure influence within the background, especially for some small areas of green disturbance, such as weeds. These interferences usually cover small areas and can, thus, be processed by an area exclusion method with a threshold of 0.05% of the image, while the minimum area of trees (with a diameter of 0.9 m, as introduced in Section 2.2) in the image of dataset 0 is 0.10%. Secondly, some jagged interferences along the edges of the RoIs might be caused by unclear boundaries resulting from shaking of the camera onboard the agricultural UAV; these jagged boundaries can be removed by expansion and erosion with a circular structure. Although a morphological treatment with a small radius could remove only small interference, the radius of expansion and erosion was set to five pixels in this paper; this radius is considered reasonable because excessive erosion and expansion would change the edges and contours, reducing the RoI extraction accuracy. Finally, the interference due to holes inside the RoIs, which could be caused by color singularities inside the fruit trees, were filled by a hole-filling algorithm.

#### 3.2.2. Chromatic Mapping Extraction Method Enhanced with MSR

Since the ERGCM considers only the *im_p_* of an image, this method is strongly sensitive to weeds. For subsequent processing, a UEJR was proposed based on the area and number of RoIs to determine whether the primary RoI result is under-extracted. When the results of the ERGCM in a weedy environment (such as environments exhibiting MWCR or LWCR conditions) are under-extracted, the weeds between RoIs might be erroneously extracted, as shown in Figure 10e,h. Following such under-extraction, independent fruit trees can be connected, significantly increasing the area of RoIs and significantly decreasing the number of independent RoIs. Let *A* denote the ratio of the total RoI area to the image area, *N* denote the number of individual RoIs in the image, *A_t_* denote the true ratio of the RoI area to the image area, and *N_t_* denote the true number of trees in an image. The UEJR could be defined based on the relationships between these factors; when *A* > *T_A_* or *N* < *T_N_*, the preliminary result is under-extracted, where *T_A_* is the threshold of the RoI area and *T_N_* is the threshold of the RoI number.

**Figure 5:**
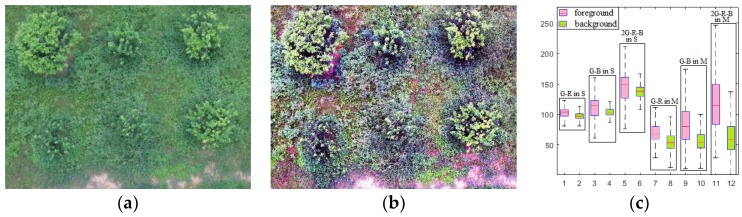
Reflection (M) and source (S) images of an example image under large weed coverage rate (LWCR) conditions and their chromatic results. (**a**)An example image under LWCR conditions of S mapping *S*_1_. (**b**) *M*_1_ of *S*_1_, processed by multi-scale retinex (MSR). (**c**) The G–R, G–B, and 2G–R–B values of the *M*_1_ of *S*_1_.

To re-extract the under-extracted images, EMSRCM based on MSR technology was proposed to reduce the influence of weeds, especially under MWCR and LWCR conditions. The MSR method was used to separate the reflection image (*M*) from the source image (*S*) by transforming the RGB image into a frequency mapping; this approach is typically used to enhance the foreground in images under darkness or fog [37,38]. Therefore, the multi-scale reflection sub-image weighted fusion method proposed by Rahman et al. and Ojala et al. [39,40] was adopted to separate *M* from *S* to further extract RoIs from *M* through chromatic aberration technology. The algorithm process is as follows: The R, G, and B color channels of images are processed with three separate scales, and the high-frequency area of fruit trees is highlighted to improve the separability between fruit trees and weeds. This algorithm can be expressed as Equation (2).

(2) $$R_{i}\left(x,y\right)=\overset{3}{\underset{j=1}{\sum }}W_{j}\left\{logS_{j}\left(x,y\right)-log\left[S_{i}\left(x,y\right)⊗G_{j}\left(x,y\right)\right]\right\},$$

where *S_i_*(*x*, *y*) is the *i*-th channel of the image, in which *i* = 1, 2, 3 correspond to the R, G, and B color channels of the image, respectively, *W_j_* is the scale weight of each channel, *W*_1_ = *W*_2_ = *W*_3_ = 1/3, ⊗ denotes a convolution operation, and *G_j_*(*x*, *y*) represents Gaussian kernel functions on different scales. Finally, the result of the *i*-th channel is $M_{i}\left(x,y\right)=\exp\left[R_{i}\left(x,y\right)\right]$. Figure 5 shows the enhanced effect of MSR on the trees against a background containing weeds. Figure 5a presents the S image of the partial example under LWCR conditions, Figure 5b presents the *M* image of *S*, and Figure 5c shows the statistical results of the tree and weed areas in the *S* and *M* images, including the G−R, G–B, and 2G–R–B values. The G–R, G–B, and 2G–R–B chromatics of the fruit trees in *S* are all close to those of the weed background, making it difficult to extract the trees directly. However, the G–R and G–B chromatics of the fruit trees in *M* are more separable than those in *S*, enhancing the separability of the trees in the 2G–R–B (the superposition of G–R and G–B) chromatic and improving the RoI extraction accuracy.

Based on these conclusions, the first step of the EMSRCM is to separate *M* from *S* and to transform *M* to a 2G–R–B mapping, thereby enhancing the trees in the weedy background. Then, a large-radius closed filter operation is applied to the image to remove small interference areas around the trees potentially attributable to some weeds in the background that have similar frequency characteristics as the citrus trees in the image. In addition, a top-hat filter is used to eliminate some interference at different positions in the local background of the image possibly caused by differences in the frequency among different background regions exposed by MSR. Then, the Otsu method and an open filter are applied to the image to extract the RoIs from the weedy background. As a large-radius morphological treatment, such as an open filter or a closed filter, is inevitably used to reduce the weedy background, the edge information of the trees is important. To minimize the loss of information during the application of the large-radius morphological method intended to reduce the influence of weeds, a convex hull transform algorithm for edge convex filling is used to improve the extraction accuracy. The entire EMSRCM process is described in Table 3.

### 3.3. Fruit Tree SVM Segmentation Model

To segment the RoIs accurately, an SVM segmentation model was established by calculating the color features and texture features of the RoIs. The color features of the RoIs include each component of the RGB, his, and La*b* color spaces, excluding the I component in the HSI color space and the L component in the La*b* color space, which represent the image brightness and are easily affected by the environment after the images are pre-processed. Therefore, the color features of the RoIs comprise the averages and variances of the R, G, B, H, S, a*, and B* components and constitute 14 dimensions in total.

For the texture features of the RoIs, the statistical texture features of the GLCM and the LBP features were adopted to reflect the regional features of the tree structure. Firstly, the GLCMs of the RoIs were calculated as *G*(*i*, *j*), and the five texture statistics of *G*(*i*, *j*) were calculated. Firstly, the contrast was calculated as $CON=\sum_{n=0}^{k-1}n^{2}\sum_{i-j=n}G\left(i,j\right)$, where k=16 indicates the gray scale of the image. *CON* measures the local change and the image matrix distribution and reflects the image clarity and texture grooving depth. A deeper grooving depth generates a greater value of *CON* and of the texture. Secondly, the energy was calculated as $ASM=\sum_{i=0}^{k}\sum_{j=0}^{k}\left(G{\left(i,j\right)}^{2}\right)$, which reflects the uniformity coefficient of the gray-level distribution and the texture thickness; a thicker texture generates a smaller energy. Thirdly, the entropy was calculated as $ENT=-\sum_{i=0}^{k}\sum_{j=1}^{k}G\left(i,j\right)lgG\left(i,j\right)$, which reflects the complexity of the image gray-level distribution; a more complex image generates a greater entropy. Fourthly, the inverse variance was calculated as $IDM=\sum_{i=0}^{k}\sum_{j=0}^{k}\frac{G\left(i,j\right)}{1+{\left(i-j\right)}^{2}}$, which reflects the local change in the image texture; a smaller change generates a larger *IDM*. Lastly, the correlation was calculated as $COR=\sum_{i=0}^{n}\sum_{j=0}^{n}\frac{\left(i,j\right)G\left(i,j\right)-u_{i}v_{j}}{s_{i}s_{j}}$, where $u_{i}=\sum_{i=0}^{k}\sum_{j=0}^{k}i\;G\left(i,j\right)$ and $v_{j}=\sum_{i=0}^{k}\sum_{j=0}^{k}jG\left(i,j\right)$, which reflects the local gray-level correlation; a stronger correlation generates a greater value of *COR*. In addition, the LBP texture feature possesses 59-dimensional distribution characteristics that can be calculated by the method introduced in Reference [41] and has the advantage of grayscale invariance.

A linear kernel-based SVM segmentation model was established based on the above 80-dimensional features, and the effect was evaluated using the following eight indicators: (1)the intersection over union (*IoU*), *IoU* $=\frac{n\left(r\right)\cap n\left(h\right)}{n\left(r\right)\cup n\left(h\right)}\times 100\%$, where *n*(*r*) is the area of tree output by the segmentation algorithm and *n*(*h*) is the manually segmented area, which represents the positioning accuracy of the image segmentation algorithm; (2) the precision of segmentation pixels (*P_I_*), *P_I_* = *TP_I_*/(*TP_I_* + *FP_I_*), which represents the correctness of the segmentation, where *TP_I_* is the true area of segmented trees, *FP_I_* is the area in which fruit trees are erroneously segmented as background, and *FN_I_* is the area where the background is erroneously segmented as fruit trees; (3) the recall rate (*R_I_*), *R_I_* = *TP_I_*/(*TP_I_* + *FN_I_*), which indicates the correctness of the segmentation of fruit trees; (4) the F1-score (*F1_I_*), *F1_I_* = 2 × *P_I_* × *R_I_*/(*P_I_* + *R_I_*), representing the general segmentation effect; (5) the correct segmentation rate of fruit trees (*CTR*) [12], which represents the segmentation accuracy of the number of trees and is used to represent the final segmentation effect in this paper; (6) the tree segmentation precision (*P_C_*, *P_C_* = *TP_C_*/(*TP_C_* + *FP_C_*), which represents the correctness of the tree segmentation accuracy, where *TP_C_* is the true number of segmented trees, *FP_C_* is the erroneously segmented number of trees when the trees are segmented into the background, and *FN_C_* is the erroneously segmented number of trees when the background is segmented into the foreground; (7) the recall rate of fruit tree segmentation (*R_C_*), *R_C_* = *TP_C_*/(*TP_C_* + *FN_C_*), which represents the effect of truly segmenting the fruit trees; (8) the F1-score of trees (*F1_C_*), *F1_C_* = 2 × *P_C_* × *R_C_*/(*P_C_* + *R_C_*), which represents the comprehensive effect of segmenting the trees.

## 4. Results and Discussion

### 4.1. Evaluation of the Influence of the Image Brightness Condition on the Segmentation Results

**Figure 6:**
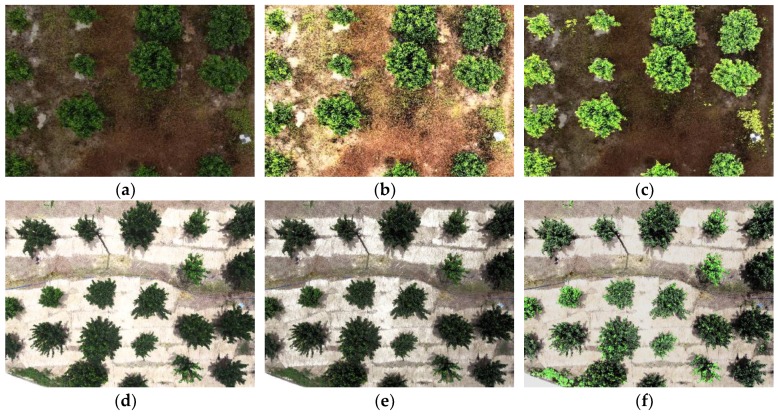
Results of two illumination compensation examples. (**a**) An example under IB conditions, *I*_1_. (**b**) *I*_1_ after applying the histogram equalization (HE). (**c**) *I*_1_ after applying the selective region intensity histogram equalization (SRIHE). (**d**) An example under IB conditions, *I*_2_. (**e**) *I*_2_ after applying the HE. (**f**) *I*_2_ after applying the SRIHE.

Figure 6 shows two example images under IB conditions and the pre-processing results of these images by the HE and SRIHE. Figure 6a shows an image with weak brightness in the foreground and background, Figure 6b shows the processed result of Figure 6a using HE, and Figure 6c shows the processed result of Figure 6a using SRIHE. Figure 6c is superior to Figure 6b, because the SRIHE takes the foreground region for illumination compensation, thereby avoiding any adjustment of the background, while the HE takes the global histogram for the adjustment. In addition, when the brightness of the foreground is lower than that of the background in an image, as shown in Figure 6d, the adjustment performed with the HE might be insufficient to effectively compensate for the foreground brightness when the background and foreground are adjusted at the same time, as displayed in Figure 6e. However, the adjustment process of the SRIHE is more relevant to the foreground, as potential foreground areas are selected for compensation; thus, the effect is better than the effect of the HE, as shown in Figure 6f. Therefore, pre-processing with the SRIHE reduces the difference in tree illumination among images to some extent and reduces the sensitivity of the algorithm to the environmental BC.

**Figure 7:**
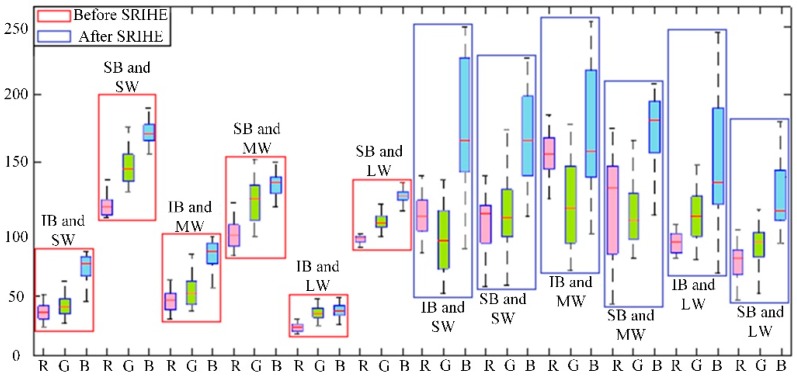
Data representation of the fruit tree areas in RGB color space before and after pre-treatment (SW, MW, and LW mean SWCR, medium weed coverage rate (MWCR), and LWCR conditions, respectively).

To evaluate the effect of applying the SRIHE, the images in the test set introduced in Section 3.2.2 were processed with the SRIHE, and their RGB components were obtained, as shown in the box plot in Figure 7. The results demonstrate that the brightness differences among the trees in different images were reduced; for example, the average values of the R components were 38 under IB and SWCR conditions and 115 under SB and SWCR conditions, but these values changed to 128 and 145, respectively, after applying the SRIHE, with the differences reduced by 60. The statistical results of other channels were also analyzed, and the standard deviation of the trees in each image band was reduced from 41 to 21. Therefore, we assert that the SRIHE reduces the difference among each component between different trees in images and makes trees more consistent under different BCs, which ensures the accuracy of the RoI extraction under different illumination environments.

A comparison of the extraction results of images under different BCs reveals that the tree segmentation method proposed in this paper is not sensitive to image brightness, as shown in Figure 11a,d. The SRIHE compensates for the illumination and reduces the variation in the influence on different images, thereby improving the extraction and segmentation accuracy. In addition, the relative chromatic aberration mapping *im_p_* used in the ERGCM and the MSR technology used in the EMSRCM are insensitive to the local brightness of images; these results are similar to the findings of Guoet al. [41] and Kyung et al. [42].

### 4.2. Evaluation of the Influence of the Weed Coverage Condition on the Segmentation

**Figure 8:**
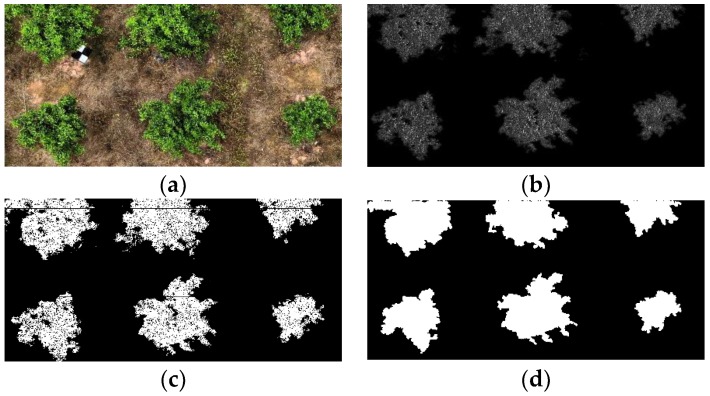
Extracted result of fruit trees in SWCR images using the RG chromatic extraction method (ERGCM). (**a**) An example SWCR image in RGB color space. (**b**) The transformed result of (**a**) using relative chromatic mapping. (**c**) Separated result of (**b**) using the Otsu method. (**d**) The result of (**c**) using morphological treatments.

In this paper, the ERGCM and EMSRCM were combined to extract RoIs, and the results show that the combination of these two methods is insensitive to natural weedy environments. The ERGCM was used to preliminarily extract the RoIs, which are suitable for the SWCR conditions of the test set, and Figure 8 shows the results. Figure 8a shows an example image in RGB color space under SWCR conditions. Figure 8b shows the transformed result mapping of Figure 8a using the relative G–R chromatic aberration mapping, which results in an obvious difference between the foreground and background. Figure 8c shows the separated result Figure 8b using the Otsu method, where the RoIs are completely separated from the image. Figure 8d shows the ERGCM result after the morphological post-treatment, in which erosion, dilation, and hole-filling operations were used to reduce the interference regions corresponding to minor misclassifications. The results demonstrate that the ERGCM is highly precise for RoI extraction and accurately extracts the edges of trees even in an SWCR background.

Because of the sensitivity of ERGCM to weeds, the UEJR was proposed based on the area and number of RoIs; thus, the values of *A*, *N*, *A_t_*, and *N_t_* in the test set were calculated. *A_t_*, *N_t_*, *A*, and *N* in the test set were extracted by the ERGCM, and *A_t_* and *N_t_* were obtained using a manual method (the manual results are regarded as a reference). The results are shown in Table 4. When comparing *A* and *N* under the same BC and different WCCs, a increased significantly, and *N* decreased significantly with an increasing weed coverage rate. For example, *A* decreased from 39.1 ± 10.2 to 1.3 ± 0.6 and *A* increased from 29 ± 8.5 to 76 ± 19.1 under IB conditions because the ERGCM is sensitive to background weed coverage. However, the true values of the area and number of RoIs (*A_t_* and *N_t_*) did not change significantly. For example, the values of *A_t_* were 30 ± 6.5, 31 ± 8.5, and 32 ± 5.8 under IB conditions with SWCR, MWCR, and LWCR backgrounds, respectively, and the corresponding values of *N_t_* were 37.7 ± 8.5, 41.7 ± 14.4, and 32.4 ± 7.1, suggesting that the true area and number of fruit trees did not change substantially. Based on this conclusion, the UEJR could be defined as follows: when *A* > *T_A_* = 45 ≈ 34 + 11.5 (%) or *N* < *T_N_* = 20 ≈ 27.8 − 7.7 (tree) for the RoIs in the image, the preliminary result is under-extracted.

**Figure 9:**
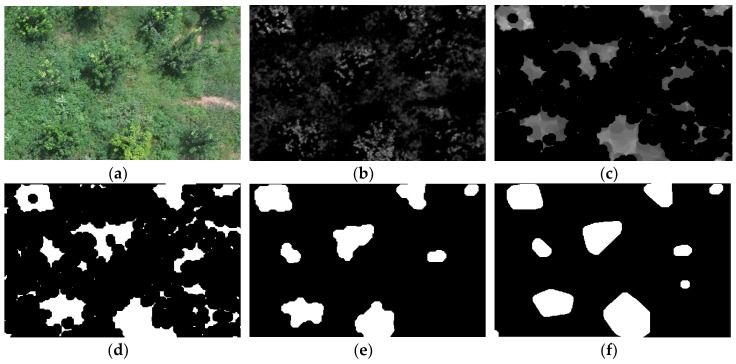
Re-extracted results of the EMSRCM. (**a**) An example image in RGB color space. (**b**) Grayscale images transformed by MSR and 2G–R–B chromatic mapping on (**a**). (**c**) Filtered result obtained by applying an open filter and a top-hat filter to (**b**). (**d**) Separated results obtained by applying the Otsu method to (**c**). (**e**) Post-processed result of (**d**) using morphological treatments. (**f**) Transformed result of (**e**) using a convex hull method.

In addition, Figure 9 shows the RoI re-extraction result with the EMSRCM, which is suitable for the under-extracted images of the ERGCM in the MWCR and LWCR images of the test set. Figure 9a shows an example image in an MWCR environment, and Figure 9b shows Figure 9a transformed to the 2G–R–B chromatic mapping after being enhanced with MSR technology. Figure 9c shows Figure 9b filtered by an open filter and a top-hat filter, revealing that interference due to the weed background is clearly decreased and that the tree area is evidently increased. Figure 9d displays Figure 9c separated by the Otsu method, which processes most of the weeds in the extracted RoIs. Figure 9e shows Figure 9d post-processed by applying the morphological treatment, which is used to exclude areas of interference. Finally, Figure 9f shows Figure 9e after applying the convex hull transform, which reduces the loss of information along the edges of the image. Therefore, the EMSRCM is an effective method for re-extracting RoIs in under-extracted images, such as the images under MWCR and LWCR conditions in the test set.

**Figure 10:**
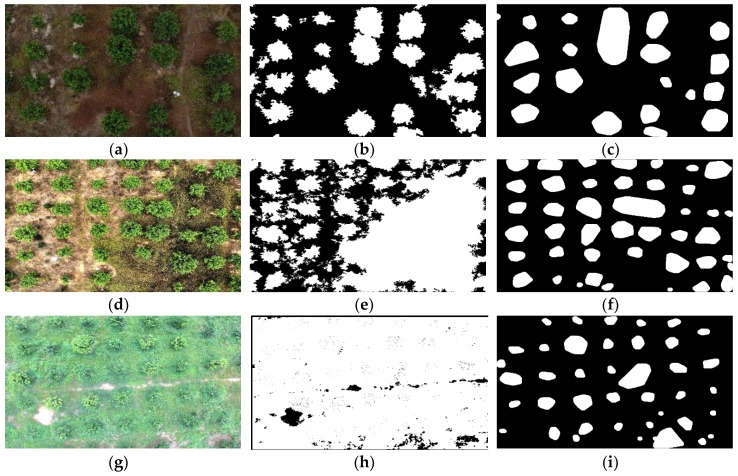
ERGCM and EMSRCM results under different WCCs. (**a**) An example image of SWCR *I_a_*. (**b**) *I_a_* processed by the ERGCM. (**c**) *I_a_* processed by the EMSRCM. (**d**) An example image of MWCR *I_b_*. (**e**) *I_b_* processed by the ERGCM. (**f**) *I_b_* processed by the EMSRCM. (**g**) An example image of LWCR *I_c_*. (**h**) *I_c_* processed by the ERGCM. (**i**) *I_c_* processed by the EMSRCM.

Figure 10 shows the RoIs extracted with the ERGCM and EMSRCM under different WCCs (including SWCR, MWCR, and LWCR conditions), which illustrate the extraction effects. Figure 10b,c show the results of the ERGCM and EMSRCM under SWCR conditions, respectively; ERGCM produces better results, as this method achieves a higher accuracy along the edges of the extracted trees. This may be because the large-radius morphology used in the EMSRCM changes the edge information in the RoIs that even the convex hull transform algorithm cannot repair. Figure 10e,f,h,i show the results of the ERGCM and EMSRCM for trees under MWCR and LWCR conditions; we found that the EMSRCM results are acceptable, whereas the ERGCM results are under-segmented in the weedy environment. In conclusion, the ERGCM and EMSRCM have unique advantages and disadvantages; for example, the ERGCM boasts high accuracy but is sensitive to the WCC, while the EMSRCM is insensitive to the presence of weeds but forfeits edge information (but is more accurate than the ERGCM). To optimize the RoI extraction effect, the proposed UEJR was used to combine the ERGCM and EMSRCM; accordingly, some images whose RoI results were judged as being under-extracted were re-extracted by the EMSRCM. Therefore, the whole extraction method is highly accurate under SWCR conditions and insensitive to weeds because the ERGCM avoids losing edge information and the EMSRCM is insensitive to weedy environments.

### 4.3. Evaluation of Fruit Tree Segmentation Results

To evaluate the fruit tree segmentation results, the training set introduced in Section 2.2 was used to train the SVM segmentation model; then, the effect of the model was tested with a test set. All the fruit trees of the test set were segmented automatically by the algorithm, and the trees were artificially marked as a reference area (the true area) to calculate the model accuracy. The evaluation of the tree segmentation accuracy included the *IoU*, which represents the correctly segmented area, and *CTR*, which represents the correct number of trees. The *P_I_*, *R_I_*, and *F1_I_* parameters of the *IoU* the *P_C_*, *R_C_*, and *F1_C_* parameters describing the *CTR* were also calculated. Table 5 shows the calculated results with and without the SVM model for the test set.

Comparing the results under IB and SWCR conditions, the *IoU* with and without the SVM model was 90.64% and 86.02%, respectively, with a 4.62% *IoU* increase (the maximum increase in *IoU* was 10.39% under IB and LWCR conditions). Similarly, to analyze the accuracy under MWCR and LWCR conditions, we know that segmentation with the SVM model can improve the segmentation accuracy. Color and texture features were used to establish the SVM model, thereby reducing the segmentation errors and improving the segmentation accuracy. However, the SVM model reduced the *R_I_* slightly and increased *P_I_*; for example, *P_I_* and *R_I_* under IB and SWCR conditions changed from 93.73% to 92.34% and from 91.71% to 98.01%, respectively, with an increase of 6.30% in *P_I_* and a reduction of 1.39% in *R_I_* (the minimum reduction in *P_I_* was 0.05% under SB and LWCR conditions, and the maximum increase in *R_I_* was 15.51% under IB and LWCR conditions). This might be because the SVM model confirms each RoI one by one and effectively excludes the erroneously segmented regions, improving *P_I_* considerably. However, the SVM model did not improve *R_I_*; rather, it reduced *R_I_* slightly when the SVM was affected by the BCs and WCCs. In summary, although the SVM model slightly reduced *R_I_*, *F1_I_* was still enhanced when *P_I_* was obviously improved. Furthermore, when comparing the results under different WCCs, the *CTR* was also reduced; for example, the *CTR* values were 90.97%, 78.38%, and 75.92% under IB and SWCR, MWCR, and LWCR conditions, respectively, without the SVM model and were changed to 92.94%, 80.71%, and 77.60%, respectively, with the SVM model. The presence of weeds in the background might affect the ERGCM and EMSRCM in the segmentation method, thereby reducing the *CTR*.

**Figure 11:**
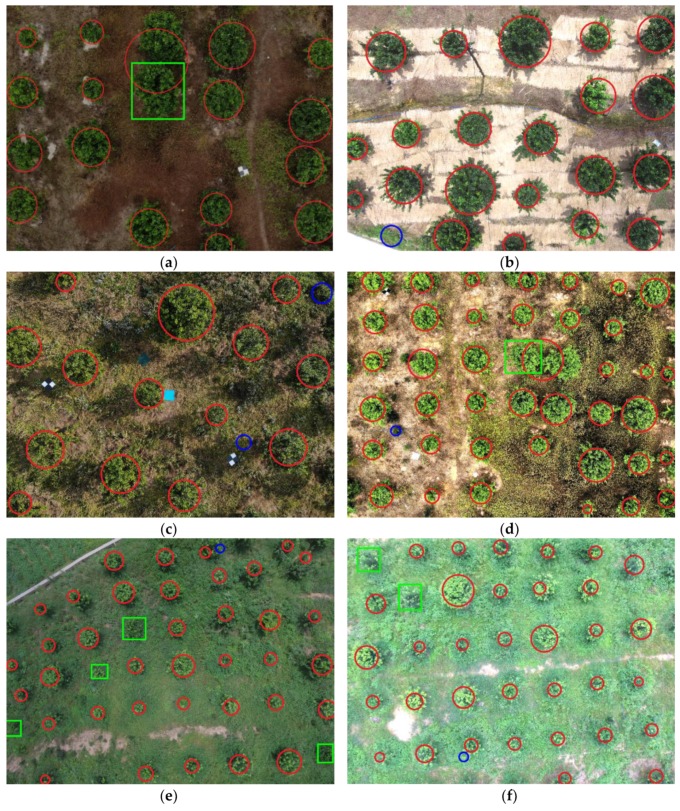
Citrus tree segmentation results for the agricultural UAV. (**a**) Segmentation results under IB and SWCR conditions. (**b**) Segmentation results under SB and SWCR conditions. (**c**) Segmentation results under IB and MWCR conditions. (**d**) Segmentation results under SB and MWCR conditions. (**e**) Segmentation results under IB and LWCR conditions. (**f**) Segmentation results under SB and LWCR conditions. The red circles represent correct results in the images, blue circles denote erroneously segmented regions, and green squares indicate missing trees.

To illustrate the final fruit tree segmentation results, Figure 11 shows examples under each condition, including different BCs and WCCs. The red circles represent correctly segmented trees in the images, where each circle center is the center of the RoI, and its area is equal to that of the segmented tree. In contrast, the blue circles denote erroneously segmented regions, and the green squares indicate missing segmented trees. Figure 9 shows that the proposed method is insensitive to the environmental brightness and the presence of weeds, as the same WCC results under IB and SB conditions verify that the illumination condition does not greatly affect the segmentation, while the results under different WCCs and the same BC show that the proposed method is applicable in weedy orchards.

Although the proposed method is weakly affected by different WCCs and BCs, there are still a few errors, such as mis-segmentations and erroneous segmentations. For example, the green rectangles of trees in Figure 11a,d are mis-segmented, indicating that a tree overlaps with another correctly segmented tree; this condition might result in a mis-segmented image. Here, the segmentation method did not divide any trees, which will be studied in the future. In Figure 11e,f, some trees are mis-segmented under LWCR conditions, possibly because the large-radius morphology treatment changed the edges considerably; thus, the small tree area affected the SVM segmentation model result. The rectangles in Figure 11b,c,f delineate erroneously segmented trees where the areas of weeds were segmented as trees, possibly because those weed areas exhibited color and texture characteristics similar to those of the trees; thus, the SVM model was affected.

**Figure 12:**
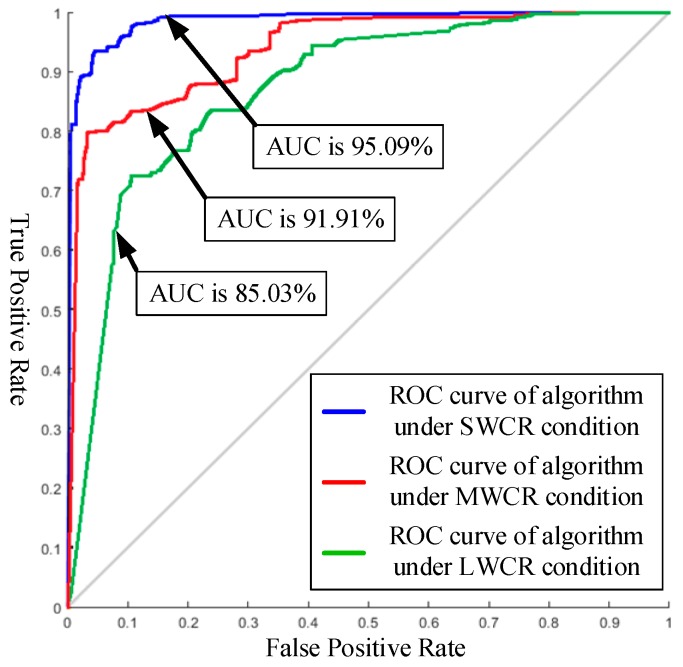
The receiver operating characteristic(ROC) curves of the support vector machine (SVM) under different WCCs on the test set.

The receiver operating characteristic (ROC) curve [43] was used to evaluate the segmentation performance of the proposed method, where the true positive rate (TPR) was plotted against the false positive rate (FPR) on different WCCs of the test set. As shown in Figure 12, the areas under the curve (AUCs) when using the resultant SVM classifier under different conditions (i.e., SWCR, MWCR, and LWCR) were 95.09%, 91.91%, and 85.03%, respectively. The results show that the SVM classifier under SWCR conditions is better than that under both MWCR and LWCR conditions. Because the color appearance of weeds is similar to that of citrus trees, the number of weeds would affect the segmentation performance; therefore, the proposed method obtained the best and worst results under SWCR and LWCR conditions, respectively. This result is consistent with the conclusion in Figure 11 and the orchard image features.

The method presented by Lin et al. [25] (method 1) and that developed by Omair et al. [22] (method 2) were used for comparison; the data used in this part were images under SB conditions from the test set. Table 6 shows that the segmented results under different WCCs were accurate for the proposed method (*CTR* = 85.27 ± 9.43), but the results of methods 1 and 2 were poor (*CTR*s = 69.49 ± 10.37 and 64.04 ± 12.82) under MWCR and LWCR conditions. The poor result of method 1 might be due to the fact that the k-means model on the a*b* plane (a* and b* components in the La*b* color space) was greatly influenced, making it difficult to segment the trees under the different WCCs. In method 2, the entropy used to describe the texture was not sufficient for the weed images in the test set, and erosion was used to delete weeds around some fruit trees, leading to the removal of some small areas of trees. In conclusion, the proposed method achieved high accuracy in the natural orchard environment studied in this paper.

## 5. Conclusions

In this paper, a citrus tree segmentation method based on monocular machine vision in a natural orchard environment was proposed, and the main conclusions were as follows.

(1) After extracting the potential foreground areas in the H component (in HSI color space) with threshold extraction technology and calculating the brightness histogram (the I component in HSI color space), the SRIHE was proposed by equalizing the brightness histogram to compensate for the foreground illumination. The results show that the proposed illumination compensation method enhances the contrast of the foreground brightness and keeps the H and S unchanged, which can compensate for weak illumination and achieve a better effect than that of traditional histogram equalization.

(2) Based on this brightness compensation process, relative RG chromatic mapping and the Otsu threshold algorithm were used to extract the RoIs of citrus trees. The results on SWCR images show that the RoI extraction accuracy of trees reached 91.66%, and the edge information was preserved.

(3) The area and number of RoIs were used to judge whether the results were under-extracted. The under-extracted images were re-extracted with the MSR enhancement algorithm and chromatic technology, and the re-extraction method was tested under MWCR and LWCR conditions; the accuracies were 79.88% and 77.27%, respectively, which indicates an insensitivity to weeds.

(4) A fruit tree SVM segmentation model was established by calculating the color and texture features of the RoIs. The segmentation results of citrus trees in natural orchard images (under different BCs and WCCs) show an average accuracy of 85.27% ±9.43%, which demonstrates that the proposed method can effectively suppress the false alarm rate.

## Figures and Tables

**Table 1:** The parameters of the unmanned aerial vehicle (UAV) and the onboard camera. CMOS—complementary metal-oxide-semiconductor; FOV—field of view; JPEG—joint photographic expert group.

System Parameter	Value
Vertical accuracy	±0.1 m
Camera sensor	1/2.3-inch CMOS
Image resolution	4032 × 3024
FOV	85°
Focal length	24 mm
Diaphragm size	f/2.8
Time of exposure	1/1000 s
Camera model	FC2103
Format/type of images	JPEG

**Table 2:** The six categories of imagedata ^1^. BC—brightness condition; WCC—weed coverage condition; IB—insufficient brightness; SB—sufficient brightness; SWCR—small weed coverage rate; MWCR—medium weed coverage rate; LWCR—large weed coverage rate.

Dataset	BC	WCC			Total
		SWCR	MWCR	LWCR	
Dataset 0	IB	64 (1344) *	29 (589)	89 (1869)	182 (3802)
	SB	57 (1351)	32 (483)	63 (1512)	152 (3346)
	Total	121 (2695)	61 (1072)	152 (3381)	334 (7148)
Training Set	IB	32 (701)	14 (222)	44 (1024)	90 (1947)
	SB	29 (641)	16 (280)	32 (780)	77 (1701)
	Total	61 (1342)	30 (502)	76 (1804)	167 (3648)
Test Set	IB	32 (643)	15 (347)	45 (845)	92 (1855)
	SB	28 (710)	16 (203)	31 (732)	75 (1645)
	Total	60(1353)	31(570)	76(1577)	167(3500)

^1^ n(m); * n is the number of images in each category, and m is the total number of fruit trees in that category.

**Table 3:** The pseudo-code of the EMSRCM (combination of multi-scale retinex (MSR) and chromatic technology). UEJR—under-extraction judgment rule; RGB—red, green, and blue; M—reflection.

Input:	The Under-Extracted Image *I_u_* Judged with the UEJR
Step 1	Separate the R, G, and B channels of *I_u_* and calculate *R_i_*by Equation (2) and *M* images (*M_u_*).
Step 2	Convert *M_u_* into the 2R-G-B chromatic mapping *I_c_*.
Step 3	Apply a closed filter *I_c_* with a 20-pixel radius to obtain *I_cc_*.
Step 4	Apply a top-hat filter *I_cc_* with a 24-pixel radius to obtain *I_cct_*.
Step 5	Apply an open filter *I_cct_* with a 20-pixel radius to obtain *I_ccto_*.
Step 6	Extract *I_cct_* with the Otsu method to obtain the binary image *I_bw_*.
Step 7	Use a convex hull transform to convert *I_bw_* into *I_cn-bw_*.
Step 8	Exclude small areas of interference in *I_cn-bw_* with a 0.05% threshold and generate *I_roi_*.
Output	The extracted foreground areas of the image, *I_roi_*.

**Table 4:** The values of *A*, *N*, *A_t_*, and *N_t_* for the test set.

BC and WCC		*A ^a^* (%)	*A_t_^b^* (%)	*N ^c^* (Trees)	*N_t_^d^* (Trees)
IB	SWCR	29 ± 8.5	30 ± 6.5	39.1 ± 10.2	37.7 ± 8.5
	MWCR	45 ± 9.1	31 ± 8.5	12.1 ± 4.4	41.7 ± 14.4
	LWCR	76 ± 19.1	32 ± 5.8	1.3 ± 0.6	32.4 ± 7.1
SB	SWCR	32 ± 7.2	31 ± 5.2	27.5 ± 17.3	25.5 ± 12.1
	MWCR	41 ± 10.9	36 ± 6.9	16.5 ± 2.7	30.1 ± 4.7
	LWCR	81 ± 17.3	38 ± 9.2	1.5 ± 0.4	35.3 ± 10.6
Average		57 ± 21.1	34 ± 11.5	17 ± 10.5	27.8 ± 7.7

*^a^ A* is the ratio of the total region of interest (RoI) area to the image area; *^b^ A_t_* is the true ratio of the RoI area to the image area; *^c^ N* is the number of individual RoIs in the image. *^d^ N_t_* is the true number of trees in the image.

**Table 5:** Statistical table of segmentation results.

Method	BC	WCC	*IoU ^a^* (%)	*P_I_^b^* (%)	*R_I_^c^* (%)	*F1_I_^d^* (%)	*CTR ^e^* (%)	*P_C_^f^* (%)	*R_C_^g^* (%)	*F1_C_^h^* (%)
Without SVM model	IB	S	86.02	93.28	91.71	92.49	90.97	97.47	93.17	95.27
		M	71.61	86.55	80.58	83.46	78.38	93.89	82.59	87.88
		L	64.89	86.52	72.19	78.71	75.92	94.22	79.63	86.31
	Average		**72.52** ± 15.34	88.90 ± 5.51	79.74 ± 13.99	84.07 ± 9.96	**81.16** ± 11.42	95.32 ± 2.80	84.53 ± 10.08	89.61 ± 6.77
	SB	S	88.19	92.98	94.47	93.72	91.66	97.78	93.60	95.65
		M	73.78	87.50	82.47	84.91	79.88	91.93	85.91	88.82
		L	68.99	87.30	75.46	80.95	77.27	92.63	82.33	87.18
	Average		**76.01** ± 14.14	89.47 ± 4.56	83.47 ± 13.69	86.37 ± 9.28	**83.09** ± 10.84	94.61 ± 4.53	87.23 ± 8.18	90.77 ± 6.36
With SVM model	IB	S	90.64	92.34	98.01	95.09	92.94	95.02	97.70	97.02
		M	77.17	84.49	89.92	87.11	80.71	93.04	85.893	87.982
		L	75.28	84.16	87.70	85.89	77.60	94.22	81.48	85.07
	Average		**80.68** ± 11.85	87.09 ± 6.55	91.64 ± 7.68	89.31 ± 7.07	**82.97** ± 11.48	94.32 ± 1.46	87.3 ± 11.89	90.10 ± 8.83
	SB	S	90.78	91.58	99.05	95.17	93.60	96.37	96.34	96.70
		M	78.32	84.69	91.25	87.84	81.02	91.09	89.32	89.51
		L	76.39	87.25	85.94	86.61	79.19	91.96	87.39	88.38
	Average		**81.85** ± 11.06	88.36 ± 5.00	91.73 ± 9.41	90.01 ± 6.55	**84.89** ± 9.43	93.62 ± 4.02	90.69 ± 6.67	91.83 ± 6.38

*^a^ IoU* is the intersection over union, which represents the positioning accuracy of the image segmentation algorithm; *^b^ P_I_* is the precision of segmentation pixels, which represents the correctness of the segmentation; *^c^ R_I_* is the recall rate of segmentation; *^d^ F1_I_* is the F1-score, representing the general segmentation effect; *^e^ CTR* is the correct segmentation rate of fruit trees and the final segmentation effect in this paper; *^f^ P_C_* is the tree segmentation precision; *^g^ R_C_* is the recall rate of fruit tree segmentation; *^h^ F1_C_* is the F1-score of tree segmentation.

**Table 6:** The effects of different segmentation methods under different WCCs.

Method		WCC			Average
		SWCR	MWCR	LWCR	
Method 1	*IoU* (%)	84.23	67.25	51.11	66.06 ± 8. 61
	*CTR* (%)	85.66	72.25	55.52	**69.49** ± 10.37
Method 2	*IoU* (%)	79.81	63.18	39.24	58.31 ± 13.72
	*CTR* (%)	80.22	70.33	48.64	**64.04** ± 12.82
Proposed method	*IoU* (%)	90.78	78.32	76.39	81.76 ± 11.06
	*CTR* (%)	93.60	81.02	79.19	**85.27** ± 9.43

## Abbreviations

The following abbreviations are used in this manuscript:

ANN	Artificial neural network
BC	Brightness condition
CNN	Convolutional neural network
*CTR*	Correct segmentation rate of fruit trees
EMSRCM	Extraction on multi-scale retinex chromatic mapping method
ERGCM	RG chromatic extraction method
GLCM	Gray-level co-occurrence matrix
HE	Histogram equalization
HSI	Hue/saturation/intensity color space
IB	Insufficient brightness
*im_p_*	Relative G–R chromatic value or mapping
*IoU*	Intersection over union
LBP	Local binary pattern
LiDar	Light detection and ranging
LWCR	Large weed coverage rate
MSR	Multi-scale retinex method
MWCR	Medium weed coverage rate
NDVI	Normalized difference vegetation index
RGB	Red/green/blue color space
RoIs	Regions of interest (potential area of fruit trees)
SB	Sufficient brightness
SfM	Structure from motion
SRIHE	Selective regions intensity histogram equalization method
SVM	Support vector machine
SWCR	Small weed coverage rate
UAV	Unmanned aerial vehicle
UEJR	Under-extraction judgement rule
WCC	Weed coverage condition
